# Exact Basilar Artery Occlusion Location Indicates Stroke Etiology and Recanalization Success in Patients Eligible for Endovascular Stroke Treatment

**DOI:** 10.1007/s00062-022-01236-0

**Published:** 2022-12-02

**Authors:** Matthias A. Mutke, Arne Potreck, Niclas Schmitt, Fatih Seker, Peter A. Ringleb, Simon Nagel, Markus A. Möhlenbruch, Martin Bendszus, Charlotte S. Weyland, Jessica Jesser

**Affiliations:** 1 Department of Neuroradiology, Heidelberg University Hospital, Im Neuenheimer Feld 400, 69120 Heidelberg, Germany; 2 Department of Neurology, Heidelberg University Hospital, Im Neuenheimer Feld 400, 69120 Heidelberg, Germany

**Keywords:** Stroke, Basilar artery occlusion, Stroke causes, Mechanical thrombectomy, Recanalization success, Clinical outcome prediction

## Abstract

**Introduction:**

Endovascular stroke treatment (EST) is commonly performed for acute basilar artery occlusion (BAO). We aimed to identify the role of the exact location of BAO in patients receiving EST regarding the stroke etiology, recanalization success and prediction of favorable clinical outcome.

**Methods:**

Retrospective analysis of 191 consecutive patients treated for BAO with EST from 01/2013 until 06/2021 in a tertiary stroke center. Groups were defined according to exact location of BAO in I: proximal third, II: middle third, III: distal third and IV: tip of the basilar artery. Univariate and multivariate analyses were performed for BAO location comparing stroke etiology, recanalization result and favorable clinical outcome according to mRS 0–3 90 days after stroke onset.

**Results:**

Occlusion sides types I–IV were evenly distributed (37, 36, 60 and 58 patients). Types I and II were more often associated with large artery atherosclerosis (50 vs. 10 patients, *p* < 0.001). Distal/tip occlusion (types III/IV) occurred mostly in cardiac embolism or embolic stroke of unknown source (89 vs. 12 in types I/II, *p* < 0.001). Occlusion site correlated with the underlying stroke etiology (AUC [Area under the curve] 0.89, *p* < 0.0001, OR [odds ratio] for embolism in type IV: 245). Recanalization rates were higher in patients with distal occlusions (type III/IV OR 3.76, CI [95% confidence interval] 1.51–9.53, *p* = 0.0076). The BAO site is not predicting favorable clinical outcome.

**Conclusion:**

The exact basilar artery occlusion site in patients eligible for endovascular stroke treatment reflects the stroke etiology and is associated with differing recanalization success but does not predict favorable clinical outcome.

**Supplementary Information:**

The online version of this article (10.1007/s00062-022-01236-0) contains supplementary material, which is available to authorized users.

## Introduction

Endovascular stroke treatment (EST) is an established therapy for both anterior circulation and posterior circulation stroke [1, 2]. Retrospective studies have reported a similar benefit of EST in the posterior circulation and in in the anterior circulation [3]. Contrarily the two available prospective studies for EST in the posterior circulation, BASICS and BEST, showed no overall benefit for EST compared to best medical treatment alone; however, both studies were limited by small cohorts, a low recruitment rate and a substantial crossover in the study group [4, 5].

Group selection to define patients who profit from EST could be based on stroke etiology. While ischemic stroke with large and medium size vessel occlusion in the anterior circulation is more often due to thromboembolism (cardiac or embolic stroke of unknown source), atherosclerotic disease with local intracranial stenosis is more often the cause of basilar artery occlusion (BAO) [6] and is associated with poor clinical outcome [7]. Patients with atherosclerotic or embolic BAO may require different treatment approaches and may have a different benefit from best medical treatment or EST.

It is so far unknown if the stroke etiology, mainly differentiating between atherosclerotic and thromboembolic occlusions, correlates with the exact BAO location. It might be beneficial to assume, if possible, the underlying stroke etiology with first angiographic imaging of the target vessel occlusion before EST.

We hypothesize that an assessment of the exact BAO location helps to identify the underlying stroke etiology (arteriosclerotic disease or embolic etiology) before EST and investigate its impact on recanalization success and clinical outcome.

## Methods

The study protocol was approved by the local ethics committee and patient informed consent was waived.

The institutional, prospectively collected, intention-to-treat stroke database was screened for patients who presented with a posterior circulation ischemic stroke due to a BAO and in whom an initial intracranial angiography was acquired between 01/2013 and 06/2021. Stroke etiology was classified into large artery atherosclerosis, cardiac embolism, other known causes (e.g. dissection), embolic stroke of unknown source (ESUS) or unknown cause.

### Classification of Occlusion Location and Recanalization Result

**Fig. 1:**
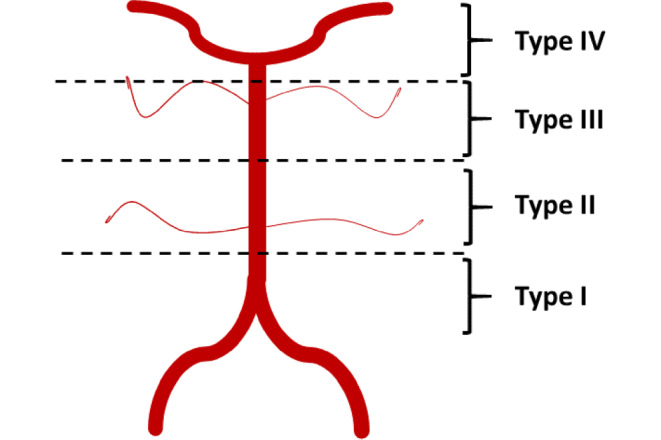
Classification of the different basilar artery occlusion locations. Anterior inferior cerebellar artery (AICA) could be occluded in either type I or type II occlusions due to the artery’s variable origin. Type I are proximal, type II middle, type III distal and type IV tip of the basilar artery occlusions

**Fig. 2:**
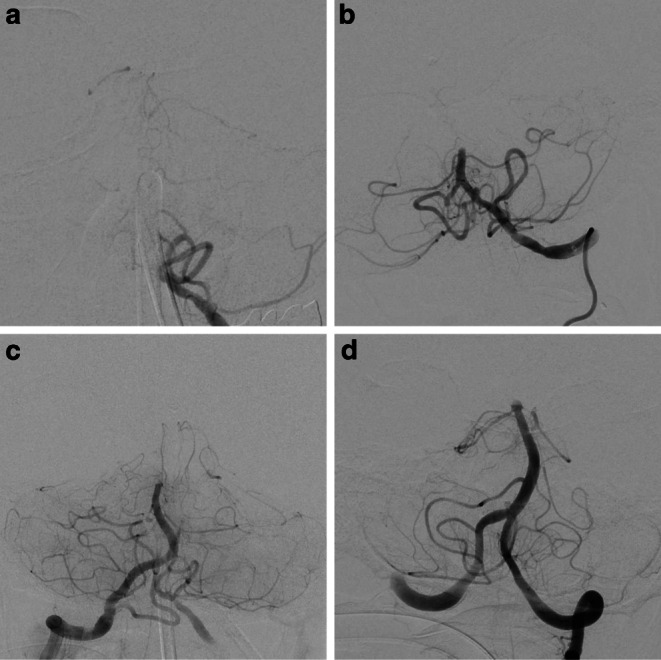
Classification of basilar artery occlusion location. Type I are proximal, type II middle, type III distal and type IV tip of the basilar artery occlusions. Exemplary images show first intracranial angiographic run during endovascular stroke treatment in four different patients with the four different occlusion locations. **a** Left vertebral artery (VA) with contrasted posterior inferior cerebellar artery (PICA) and occlusion at basilar artery (BA) root. **b** Left VA with PICA and proximal occlusion of BA directly distal to AICA origin. **c** Right vertebral artery with contrasted PICA, contralateral V4 segment of VA and BA until occlusion proximal to superior cerebellar arteries (SuCA). **d** Left VA with contrasted contralateral V4 segment of VA, BA with occlusion distal to SuCA origin

Basilar artery occlusion location was determined on initial angiogram of the target vessel occlusion and classified into four categories: proximal third (I), middle third (II), distal third (III) and tip of the basilar artery (IV) (Fig. 1). Distal third (III) occlusions were defined as occlusions distal to the anterior inferior cerebellar artery (AICA) partial or complete occlusion of the superior cerebellar arteries (SUCAs). Tip of the basilar artery (IV) occlusions were defined as occlusions distal to the superior cerebellar arteries (SuCA) and complete or partial occlusions of the junction into the posterior cerebral artery (PCA), for examples see Fig. 2. Angiography images for occlusion location and recanalization result were read by two experienced neuroradiologists (MM with 7 years and CW with 5 years experience in stroke imaging). In the case of disagreement, a consensus was reached.

The final recanalization result was rated as failed target vessel recanalization with remaining BAO or proximal branch occlusion leaving a >50% filling defect in the target vessel downstream territory in angiographic imaging (TICI 0–2a) or successful target vessel recanalization with complete reperfusion of the basilar artery without remaining proximal basilar artery branch occlusion (TICI 2b–3) [8].

### Performance of EST for Basilar Artery Occlusion

The decision for EST was made by consensus between the neurologist and the neurointerventionalist after initial stroke imaging with CT or MRI. Intravenous thrombolysis was administered according to national and international guidelines. The choice of the sedation mode in the complete study cohort was made according to the patient’s compliance, severity of the stroke syndrome and level of consciousness. In the standard approach for EST, a transfemoral access is conducted followed by placing a guide catheter in the subclavian artery (7F/80 cm Flexor Shuttle, Cook Medical, Bloomington, IN, USA). Subsequently, a distal access catheter is introduced to the vertebral artery (since 2013 Sofia 5F, MicroVention, Aliso Viejo, CA, USA). The first line approach (performing direct thrombus aspiration or stent-retriever thrombectomy in combination with continuous distal aspiration using a distal aspiration catheter), as well as the choice of material used for EST was at the discretion of the treating neurointerventionalist. The following stent-retriever types were used most often since 2013 in descending order of frequency: Solitaire (Medtronic, Dublin, Ireland), Trevo (Stryker Neurovascular, Kalamazoo, MI, USA), pRESET (phenox, Bochum, Germany). At the study facility, EST for BAO has been performed as an emergency treatment option since earlier than 2012. Patients included in 2013 until 2015, before the indication for EST went from expert opinion to first-line treatment for the anterior circulation, were all treated in the acute phase of ischemic stroke. Over the years, more patients were treated with EST for BAO in the study facility, which is reflected by the number of patients included per year (2013–2015: 13/year, 2016–2018: 26/year, 2019/2020: 30/year).

### Inclusion and Exclusion Criteria

All patients with acute stroke symptoms and indications for EST due to acute BAO on preinterventional imaging were assessed for this study. Exclusion criteria were spontaneous recanalization of the target vessel occlusion and failure of angiographic assessment of the target vessel occlusion due to failed vascular access to the vertebral artery.

Stroke etiology was classified according to diagnostic work-up at least containing 24 h ECG, transthoracic or transesophageal echocardiography and neuroduplex sonography, charted with medical reports post-EST and evaluated by a board-certified stroke neurologist. The ESUS criteria were used according to the definition proposed by the International Cryptogenic Stroke/ESUS Working Group, which requires the absence of high-risk or intermediate-risk cardiovascular causes [9]. Patients with incomplete stroke etiology assessment due to fatality shortly after EST were classified as unknown etiology. For the outcome analysis patients with missing mRS 90 days after stroke onset were excluded.

### Study Endpoints

The three study endpoints were favorable clinical outcome 90 days after stroke onset measured by the modified Rankin scale (mRS 0–3), stroke etiology and recanalization success of the basilar artery as target vessel. The recanalization result was also evaluated as a function of the thrombectomy technique (contact aspiration vs. stent retriever thrombectomy under continuous aspiration).

### Statistical Analysis

Statistical analysis was performed with GraphPad PRISM Version 9.0.1 (GraphPad Software, San Diego, CA, USA). Normality tests were performed, and statistical tests chosen accordingly. Patients were grouped according to the location of BA occlusion.

Groups were compared for differences in stroke etiology, clinical outcome after 90 days and thrombectomy technique with a Kruskal-Wallis test with Dunn’s correction for multiple comparisons or the Mann-Whitney U test when combining groups and comparing two groups only. Frequency of successful recanalization, type of first thrombectomy attempt and frequency of stenting across different occlusion groups were compared with a χ^2^-test for multiple groups or Fisher’s exact test for two groups in case groups were combined. Also, the predictive value of the occlusion location for stroke etiology was assessed with univariate logistic regression analyses. As dichotomized outcome parameters, patients were grouped with cardiac embolism and embolic stroke of unknown source versus patients with large artery atherosclerosis. Patients with other causes or unknown causes were excluded from the analysis. The significance level was set to *p* = 0.05. Medians are given with their interquartile range (IQR). All confidence intervals are quoted as 95% confidence interval (CI). The predictive value for each clinical variable (see table 2 in the supplement) was assessed with a univariate logistic regression analysis with favorable outcome (mRS 0–3 at 90 days) as outcome variable. Significant predictive variables were included in a multiple logistic regression model.

## Results

All patients (*n* = 212) with indications of EST due to BAO on preinterventional imaging were assessed for this study (also see the flowchart in figure 1 of the supplement) and 10 patients with spontaneous recanalization of the target vessel occlusion after groin puncture (after i.v. thrombolysis) were excluded. Also, 11 patients with no angiographic assessment of the target vessel occlusion due to failed vascular access to the vertebral artery were excluded.

A total of 191 patients were included in the final analysis. For 174 patients, mRS after 3 months was available. Patients’ demographic and clinical characteristics are provided in Table 1.

**Table 1:** Stroke patients’ demographic, clinical, imaging and treatment characteristics, stroke etiology and outcome. Type I are proximal, type II middle, type III distal and type IV tip of the basilar artery occlusions

Basilar occlusion type		*n* = 191	*n* = 37	*n* = 36	*n* = 60	*n* = 58
		All	Type I	Type II	Type III	Type IV
Stroke patient demographic characteristics	Age (years), median (IQR)	**76 (68–82)**	*69 (59–79)*	*79 (73–84)*	*78 (67–84)*	*76 (71–82)*
	Male, *n* (%)	**107 (56)**	*19 (51)*	*25 (69)*	*31 (52)*	*32 (55)*
	Coronary heart disease, *n* (%); m.v. 8	**57 (31)**	*6 (19)*	*8 (24)*	*22 (37)*	*21 (37)*
	Arterial hypertension, *n* (%); m.v. 5	**157 (84)**	*28 (80)*	*30 (86)*	*51 (85)*	*48 (84)*
	Known atrial fibrillation, *n* (%); m.v. 7	**74 (40)**	*7 (21)*	*11 (32)*	*25 (42)*	*31 (54)*
	Diabetes type II, *n* (%); m.v. 5	**42 (23)**	*4 (12)*	*10 (29)*	*12 (20)*	*16 (28)*
	Dyslipidemia, *n* (%); m.v. 8	**60 (33)**	*9 (27)*	*12 (35)*	*19 (32)*	*20 (36)*
	Prestroke mRS, median (IQR); m.v. 3	**1 (0–2)**	*0 (0–2)*	*1 (0–2)*	*1 (0–2)*	*1 (0–2)*
Stroke clinical and imaging characteristics	Wake-up stroke, *n* (%)	**74 (39)**	*19 (51)*	*15 (42)*	*25 (42)*	*15 (26)*
	Transfer from another hospital, *n* (%)	**108 (57)**	*24 (65)*	*24 (67)*	*31 (52)*	*29 (50)*
	NIHSS at admission, median (IQR); m.v. 2	**21 (9–35)**	*20 (10–31)*	*27 (11–37)*	*22 (9–35)*	*18 (8–31)*
	pcASPECTS on first imaging (CT or MRI); m.v. 7	**9 (7–10)**	*8 (7–9)*	*9 (8–10)*	*9 (7–10)*	*10 (8–10)*
Stroke treatment characteristics	i.v. thrombolysis, *n* (%); m.v. 5	**84 (45)**	*14 (38)*	*15 (41)*	*26 (45)*	*29 (50)*
	Time from symptom onset to groin puncture (min), median (IQR)	**360 (221–722)**	*564 (348–1339)*	*350 (222–525)*	*375 (281–743)*	*257 (161–417)*
	Time from symptom onset to final recanalization (min), median (IQR)	**440 (300–807)**	*660 (398–1440)*	*442 (357–741)*	*472 (344–838)*	*342 (223–487)*
	First thrombectomy attempt: stent retriever, *n* (%)	**132 (69)**	*17 (46)*	*27 (75)*	*50 (83)*	*38 (66)*
	First thrombectomy attempt: aspiration, *n* (%)	**38 (20)**	*6 (16)*	*5 (14)*	*8 (13)*	*19 (33)*
	First thrombectomy attempt: stenting, *n* (%)	**9 (5)**	*6 (16)*	*1 (3)*	*1 (2)*	*1 (2)*
	First thrombectomy attempt: other, *n* (%)	**3 (2)**	*2 (5)*	*1 (3)*	*0*	*0*
	Occlusion not reached, *n* (%)	**9 (5)**	*6 (16)*	*2 (6)*	*1 (2)*	*0*
	Number of thrombectomy maneuvers per intervention, median (IQR)	**2 (1–3)**	*1 (0–3)*	*2 (1–3)*	*2 (1–3)*	*2 (1–3)*
	Intracranial stenting, *n* (%)	**40 (21)**	*16 (43)*	*15 (42)*	*7 (12)*	*2 (3)*
	Successful vessel recanalization, *n* (%)	**170 (89)**	*30 (81)*	*29 (81)*	*55 (92)*	*56 (97)*
Stroke etiology	Atherosclerosis, *n* (%)	**60 (31)**	*25 (68)*	*25 (69)*	*9 (15)*	*1 (2)*
	Cardiac embolism, *n* (%)	**67 (35)**	*1 (3)*	*7 (19)*	*24 (40)*	*35 (60)*
	Other causes, *n* (%)	**5 (3)**	*2 (5)*	*1 (3)*	*1 (2)*	*1 (2)*
	ESUS, *n* (%)	**34 (18)**	*4 (11)*	*0*	*16 (27)*	*14 (24)*
	Unknown, *n* (%)	**25 (13)**	*5 (14)*	*3 (8)*	*10 (17)*	*7 (12)*
Stroke outcome	mRS at discharge: 6 (= early fatality), *n* (%); m.v. 16	**61 (35)**	*12 (38)*	*18 (53)*	*17 (31)*	*14 (25)*
	mRS after 90 days: 0–3, *n* (%); m.v. 17	**55 (31)**	*8 (27)*	*8 (23)*	*19 (33)*	*20 (38)*

*n* number of patients, *m.v.* missing values for all patients, *i.v.* intravenous, *IQR* interquartile range, *mRS* modified Rankin scale, *NIHSS* National Institute of Health stroke scale, *pcASPECTS* posterior circulation Alberta stroke programme early CT score, *ESUS* embolic stroke of undetermined source

A proximal occlusion (type I) was found in 37 (20%), middle (type II) in 36 (19%), distal (type III) in 60 (31%) and a basilar tip occlusion (type IV) in 58 (30%) patients. Characteristics for different occlusion locations are given in Table 1.

### Stroke Etiology According to Basilar Artery Occlusion Site

**Fig. 3:**
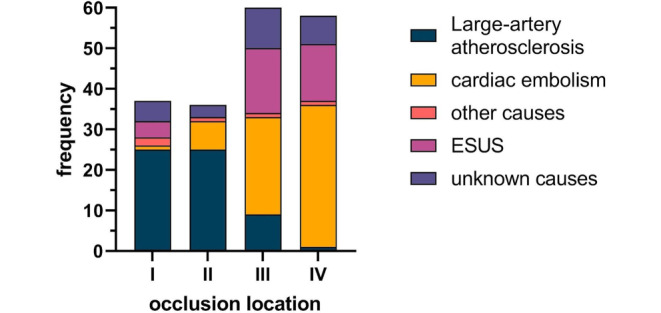
Distribution of stroke etiology across four different basilar artery occlusion locations. *ESUS* embolic stroke of unknown source. Type I are proximal, type II middle, type III distal and type IV tip of the basilar artery occlusions

Stroke etiology was large artery atherosclerosis in 60 (31%), cardiac embolism in 67 (35%), other causes in 5 (3%), ESUS in 34 (18%) and unknown etiology in 25 patients (13%). The 13 patients with early fatality (death within the first 72 h) and incomplete stroke etiology assessment, were classified as unknown etiology. Prevalence of stroke etiology as a function of the occlusion location is given in Fig. 3.

In types I and II occlusions, large artery atherosclerosis was more frequent compared to distal and tip occlusions (50 vs. 10 cases, respectively, *p* < 0.001). In distal and tip occlusion (types III and IV), there were more patients with cardiac embolism or ESUS compared to proximal and middle occlusions (89 vs. 12 cases, *p* < 0.001, see Table 1).

Stroke etiology differed between the four BAO locations (*p* < 0.0001), in particular between types I and III (*p* = 0.0005), types I and IV (*p* = 0.0002) as well as between types II and III (*p* < 0.0001) and types II and IV (*p* < 0.0001). The complete results of the multiple comparison analysis are provided in the supplement (table 1).

In logistic regression analysis, type I occlusions were highly predictive for large artery atherosclerosis whereas embolism was seldom (OR [odds ratio] 0.2, CI [95% confidence interval] 0.1–0.5, *p* = 0.0010). Types III and IV occlusions were highly predictive for an embolic cause (OR 22.2, CI 7.2–82, *p* < 0.0001 in type III and OR 245, CI 39.8–4882, *p* < 0.0001 in type IV occlusions). The AUC (area under the curve) indicates a high association of BAO site and stroke etiology with 0.89 (CI 0.84 to 0.95, *p* < 0.0001). The ROC curve is provided in the supplement (figure 2).

### Recanalization Success According to Basilar Artery Occlusion Site

In occlusion type I, target vessel recanalization was achieved in 30 (81%), in type II in 29 (81%), in type III in 55 (92%) and in type IV in 56 patients (97%). Type I and II occlusions had a lower recanalization rate compared to types III and IV (OR for types III and IV: 3.76, CI 1.51–9.53, *p* = 0.0076). Overall, recanalization of the basilar artery with EST failed in *n* = 21 patients (11%).

First thrombectomy attempt was performed with a stent retriever in 132/191 patients (69%) overall. Compared to contact aspiration it was the preferred thrombectomy technique in occlusion types II, III and IV. In type I occlusions it was used in 17/37 (46%), in type II in 27/36 (75%), in type III in 50/60 (83%) and in type IV in 38/58 (65%). In 38/191 (20%) patients, aspiration was performed first, with a majority in type IV occlusions in 19/58 (33%) patients. It was performed first in type I in 6/37 (16%), in type II in 5/36 (14%) and in type III in 8/60 (14%) patients.

Comparing primary contact aspiration and stent-retriever thrombectomy there was no difference in recanalization result between different occlusion types (*p* = 0.0595).

In 21 patients (11%), no thrombectomy maneuver was performed. Of those, 9 underwent direct stenting, the majority in type I with 6/37 (16%) and 1 patient each in types II, III and IV. One patient with type I occlusion underwent direct balloon angioplasty and two patients with a type I and a type II occlusion respectively underwent intra-arterial thrombolysis. In the remaining 9 patients, the occluded basilar artery could not be reached for therapeutic intervention, mostly in type I occlusions with 6/37 patients (16%), in type II in 2 patients (5%) and in type III in 1 patient only.

Median number of thrombectomy attempts (stent retriever or aspiration) was 2 (IQR 1–3). There was no difference in the number of thrombectomy attempts between the four different occlusion locations (*p* = 0.0601).

Of the patients 40 (21%) underwent intracranial stenting, 16 (43%) in type I, 15 (42%) in type II, 7 (12%) in type III and 2 (3%) in type IV. The OR for stenting in type I or II occlusions was 8.9 (CI 3.8–21.1) and 0.11 for type III or IV occlusions (CI 0.05–0.26, *p* < 0.0001).

### Prediction of Favorable Clinical Outcome According to Basilar Artery Occlusion Site

For this analysis we included 174 patients, as for 17 of the 191 patients included in the study mRS at 90 days after stroke treatment was not available. There was no difference in favorable clinical outcome (mRS 0–3) between the four different occlusion locations (*p* = 0.0929, see figure 3 in the supplement). Favorable clinical outcome was 27% in type I, 23% in type II, 33% in type III and 38% in type IV across occlusion locations. Significant predictors for favorable outcome in the univariate regression analysis were age (OR 0.7647, CI 0.4004–1.458, *p* = 0.4144), NIHSS at admission (OR 0.9304, CI 0.9021–0.9572, *p* < 0.0001), prestroke mRS (OR 0.6623, CI 0.4845–0.8826, *p* = 0.0068) and wake-up stroke (OR 0.429, CI 0.2026 to 0.8647, *p* = 0.0215, see table 2 in the supplement). In the subsequent multivariate logistic regression model, NIHSS at admission (OR 0.93, CI 0.90–0.96, *p* < 0.0001) and wake-up stroke (OR 0.35, CI 0.15–0.76, *p* = 0.0103) remained significant.

## Discussion

This single-center retrospective analysis of 191 patients consecutively treated with endovascular stroke therapy (EST) for basilar artery occlusion (BAO) shows that stroke etiology and recanalization success correlate with the particular BAO location. Thus, by visualization of the target vessel occlusion, the stroke etiology defined later after the neurologic diagnostic work-up following acute treatment can already be assumed.

This finding allows the interventionalist to plan ahead after initial imaging of the target vessel occlusion during EST of the posterior circulation: Distal or tip BAO mostly due to embolic occlusions will not require stenting and recanalization success is more likely. Contrarily, proximal BAOs are very unlikely caused by embolism in this study (OR 0.2) and more often atherosclerotic. In middle BAO most patients had atherosclerotic disease, however, in this location with a significant overlap with embolic etiology as well. Both proximal and middle BAO were less likely to be successfully recanalized and required more often stent-assisted PTA.

Lee et al. [7] and Kim et al. [10] showed that concerning angiographic imaging only, atherosclerotic BAO is more likely to be proximal and nonatherosclerotic BAO is more likely to be distal. The mentioned small cohort studies defined vessel stenosis as atherosclerotic disease and did not further include the post-stroke diagnostics. In this study, we determined etiology according to the TOAST classification [11] after full neurologic work-up including cardiovascular diagnostics. Therefore, our results allow a classification of etiology according to the first angiographic image with higher diagnostic confidence.

The finding that proximal BA occlusions are more difficult to recanalize is in line with Baik et al. [12] who found a better recanalization rate in patients with embolism without vertebral artery stenosis (29 out of 34 patients, 85% successful recanalization) compared to patients having in situ atherosclerotic thrombosis (11 of 20 patients, 55% successful recanalization). Kim et al. [10] reported a recanalization rate of 89.5% in 19 patients with atherosclerotic disease which is similar to our results (81% in proximal and middle occlusions); however, a 100% successful recanalization in patients with atherosclerotic disease was reported by Lee et al. [7] in 15 and Gao et al. [13] in 13 patients. This highlights potential heterogeneity between centers with different treatment approaches and patient selection with different baseline characteristics and further warrants a uniform pretherapeutic assessment of potential stroke etiology.

In our study, the BAO site did not predict favorable outcome. While previous studies did not correlate occlusion location directly with clinical outcome, clinical outcome was found to be independent from stroke etiology in most studies before (Wu et al. [14], Katsumata et al. [15], Lee et al. [7]). Kim et al. [10] observed a slightly higher OR for unfavorable outcome in patients with atherosclerotic disease, which might again be attributed to different approaches in rescue treatment. On the other hand, Lee et al. [16] observed a poorer outcome in patients with atherosclerotic disease which may reflect patient heterogeneity and smaller patient samples in single centers.

The study results might also have implications for published and ongoing trials on posterior circulation EST. Alawieh et al. report from the STAR collaboration that stent-retriever thrombectomy and combined approaches are associated with a lower OR for favorable clinical outcome compared to aspiration alone in posterior circulation EST. The authors did not report the exact BAO location, but with the knowledge of distal BAO occlusion being more likely due to embolic events, this could be attributed to a subgroup effect if more patients with distal occlusions were included [17].

The two prospective studies comparing EST for BAO with best medical treatment, BASICS [4] and BEST [5], did not report on the exact occlusion location within the study groups, information that might be necessary for evaluating the study results in the light of the different recanalization results and stroke etiology as shown in this study.

### Limitations

This is a retrospective single center study. Limitations of this study therefore concern a preselection of patients for endovascular treatment made according to local and national guidance as well as local preferences regarding treatment strategies. The distribution of intracranial atherosclerotic disease also varies according to ethnicities and populational exposure to risk factors. While the frequency of atherosclerotic disease alone might not influence these study results, the pattern of atherosclerotic lesions might differ between cohorts.

## Conclusion

In patients with basilar artery occlusion eligible for endovascular stroke treatment, the exact occlusion site reflects the stroke etiology with atherosclerotic occlusions being proximal and thromboembolic occlusions being distal. Distal occlusions have a higher chance for successful target vessel recanalization. The occlusion site does not predict favorable clinical outcome.

## Supplementary Information

Fig. 1. Flowchart of study inclusion and exclusion; Fig. 2. ROC curve occlusion location predicts cardiac embolism or ESUS (embolic stroke of unknown source). Logistic regression model with AUC = 0.89 (*p* < 0.0001); Fig. 3. Distribution of clinical outcome 90 days post-stroke on the modified Rankin scale (mRS) according to basilar artery occlusion type. Type I are proximal, type II middle, type III distal and type IV tip of the basilar artery occlusions; Table 1. Dunn’s multiple comparisons test of differences in stroke etiology; Table 2. Results of the binary univariate logistic regression analysis of favorable outcome including clinical variables.

## Abbreviations

AICA: Anterior inferior cerebellar artery
AUC: Area under the curve
BAO: Basilar artery occlusion (CI: 95% confidence interval)
CI: 95% confidence interval
CT: Computed tomography
ECG: Electrocardiogram
EST: Endovascular stroke treatment
ESUS: Embolic stroke of unknown source
IQR: Interquartile range
MRI: Magnetic resonance imaging
mRS: Modified Rankin scale
NIHSS: National Institutes of Health Stroke Scale
OR: Odds ratio
PCA: Posterior cerebral artery
pcASPECTS: Posterior circulation Alberta stroke program early CT score
ROC: Receiver operating characteristics
STAR: Stroke Thrombectomy and Aneurysm Registry
SUCA: Superior cerebellar artery
TOAST: Trial of Org 10172 in Acute Stroke Treatment

## References

[1] Meinel TR, Kaesmacher J, Chaloulos-Iakovidis P (2019). Mechanical thrombectomy for basilar artery occlusion: efficacy, outcomes, and futile recanalization in comparison with the anterior circulation. J Neurointervent Surg.

[2] Goyal M, Menon BK, van Zwam WH (2016). Endovascular thrombectomy after large-vessel ischaemic stroke: a&nbsp;meta-analysis of individual patient data from five randomised trials. Lancet.

[3] Weber R, Minnerup J, Nordmeyer H (2019). Thrombectomy in posterior circulation stroke: differences in procedures and outcome compared to anterior circulation stroke in the prospective multicentre REVASK registry. Eur J Neurol.

[4] Schonewille WJ, Wijman CAC, Michel P (2009). Treatment and outcomes of acute basilar artery occlusion in the Basilar Artery International Cooperation Study (BASICS): a&nbsp;prospective registry study. Lancet Neurol.

[5] Liu X, Dai Q, Ye R (2020). Endovascular treatment versus standard medical treatment for vertebrobasilar artery occlusion (BEST): an open-label, randomised controlled trial. Lancet Neurol.

[6] Weyland CS, Neuberger U, Potreck A (2021). Reasons for failed mechanical thrombectomy in posterior circulation ischemic stroke patients. Clin Neuroradiol.

[7] Lee YY, Yoon W, Kim SK (2017). Acute basilar artery occlusion: differences in characteristics and outcomes after endovascular therapy between patients with and without underlying severe atherosclerotic Stenosis. Am J Neuroradiol.

[8] Zaidat OO, Yoo AJ, Khatri P, Tomsick TA, von Kummer R, Saver JL (2013). Recommendations on angiographic revascularization grading standards for acute ischemic stroke: a&nbsp;consensus statement. Stroke.

[9] Hart RG, Diener HC, Coutts SB (2014). Embolic strokes of undetermined source: the case for a&nbsp;new clinical construct. Lancet Neurol.

[10] Kim YW, Hong JM, Park DG (2016). Effect of intracranial atherosclerotic disease on endovascular treatment for patients with acute vertebrobasilar occlusion. Am J Neuroradiol.

[11] Adams HP, Bendixen BH, Kappelle LJ (1993). Classification of subtype of acute ischemic stroke. Definitions for use in a&nbsp;multicenter clinical trial. TOAST. Trial of Org 10172 in Acute Stroke Treatment. Stroke.

[12] Baik SH, Park HJ, Kim JH, Jang CK, Kim BM, Kim DJ (2019). Mechanical thrombectomy in subtypes of basilar artery occlusion: relationship to recanalization rate and clinical outcome. Radiology.

[13] Gao F, Lo WT, Sun X, Mo DP, Ma N, Miao ZR (2015). Combined use of mechanical thrombectomy with angioplasty and stenting for acute basilar occlusions with underlying severe Intracranial vertebrobasilar Stenosis: preliminary experience from a&nbsp;single Chinese center. AJNR Am J Neuroradiol.

[14] Wu L, Rajah GB, Cosky EE (2021). Outcomes in endovascular therapy for basilar artery occlusion: intracranial atherosclerotic disease vs. embolism. Aging Dis.

[15] Katsumata M, Ota T, Tsuruta W (2021). Comparisons of characteristics and outcomes after mechanical thrombectomy for vertebrobasilar occlusion with cardioembolism or atherosclerotic brain infarction: data from the tokyo-Tama-registry of acute Endovascular Thrombectomy (TREAT). World Neurosurg.

[16] Lee WJ, Jung KH, Ryu YJ (2018). Impact of stroke mechanism in acute basilar occlusion with reperfusion therapy. Ann Clin Transl Neurol.

[17] Alawieh AM, Eid M, Anadani M (2020). Thrombectomy technique predicts outcome in posterior circulation stroke-insights from the STAR collaboration. Neurosurgery.

