# Supplementary material for: Exact Basilar Artery Occlusion Location Indicates Stroke Etiology and Recanalization Success in Patients Eligible for Endovascular Stroke Treatment
Source: Clin Neuroradiol. 2022 Dec 2;33(2):483–90. doi: 10.1007/s00062-022-01236-0 (PMC10219858; doi:10.1007/s00062-022-01236-0)
Supplement: Supplementary file 1 — Fig. 1. Flowchart of study inclusion and exclusion; Fig. 2. ROC curve occlusion location predicts cardiac embolism or ESUS (embolic stroke of unknown source). Logistic regression model with AUC = 0.89 (p < 0.0001); Fig. 3. Distribution of clinical outcome 90 days post-stroke on the modified Rankin scale (mRS) according to basilar artery occlusion type. Type I are proximal, type II middle, type III distal and type IV tip of the basilar artery occlusions; Table 1. Dunn’s multiple comparisons test of differences in stroke etiology; Table 2. Results of the binary univariate logistic regression analysis of favorable outcome including clinical variables. [file 62_2022_1236_MOESM1_ESM.docx]

**Supplemental material**


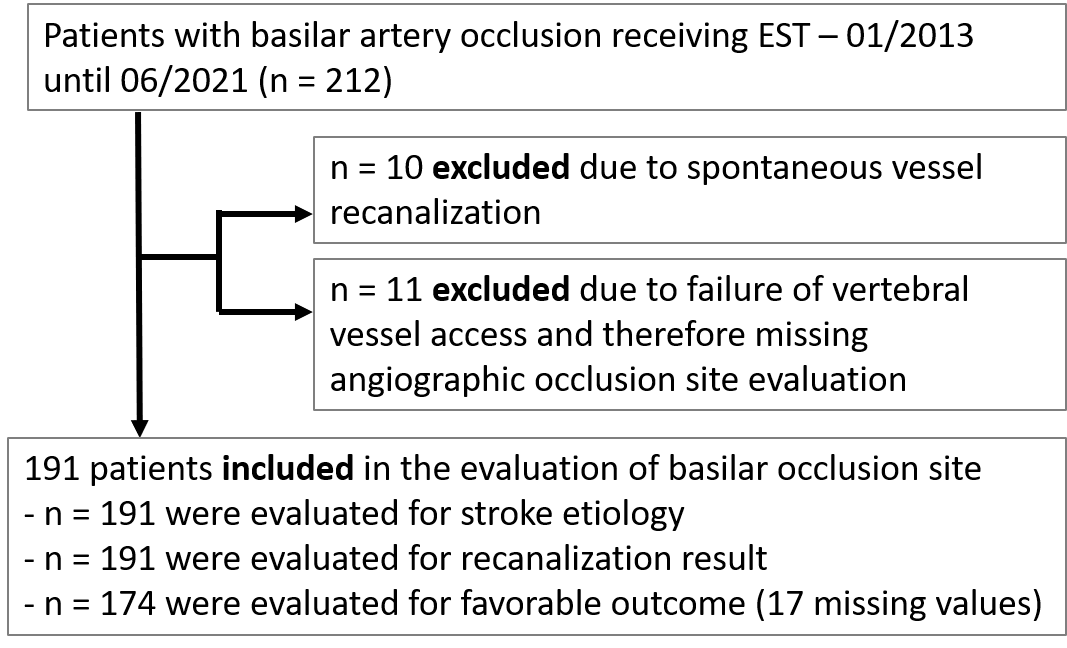


**Figure 1. Flowchart of Study Inclusion and Exclusion**

**
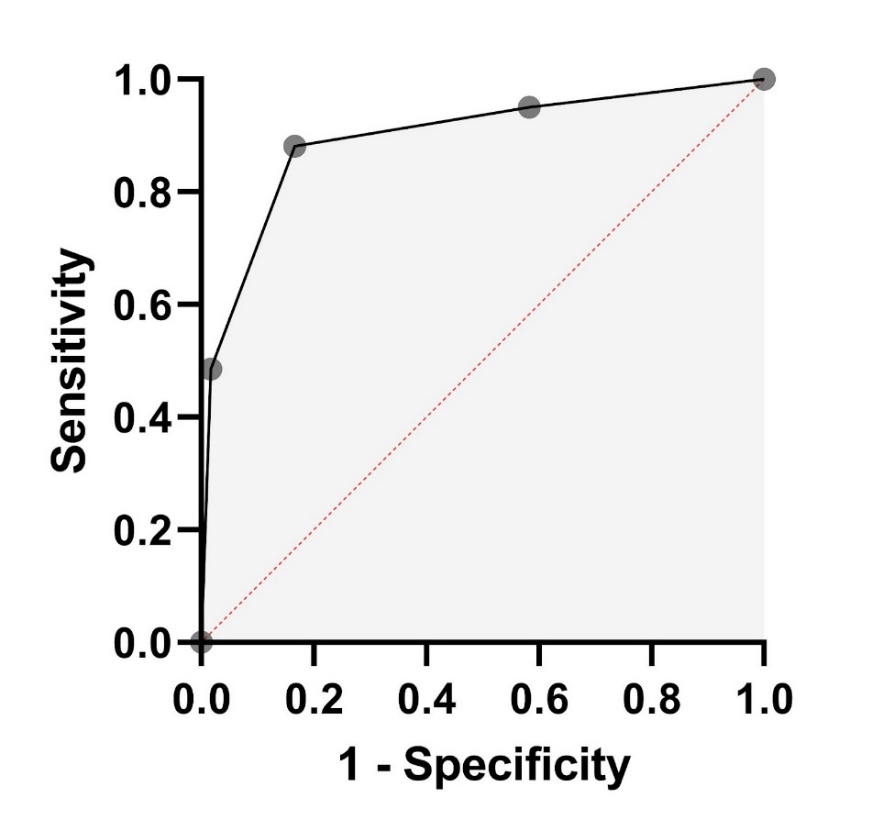
**

**Figure 2. ROC curve** Occlusion location predicts cardiac embolism or ESUS (embolic stroke of unknown source). Logistic regression model with AUC = 0.89 (p<0.0001).


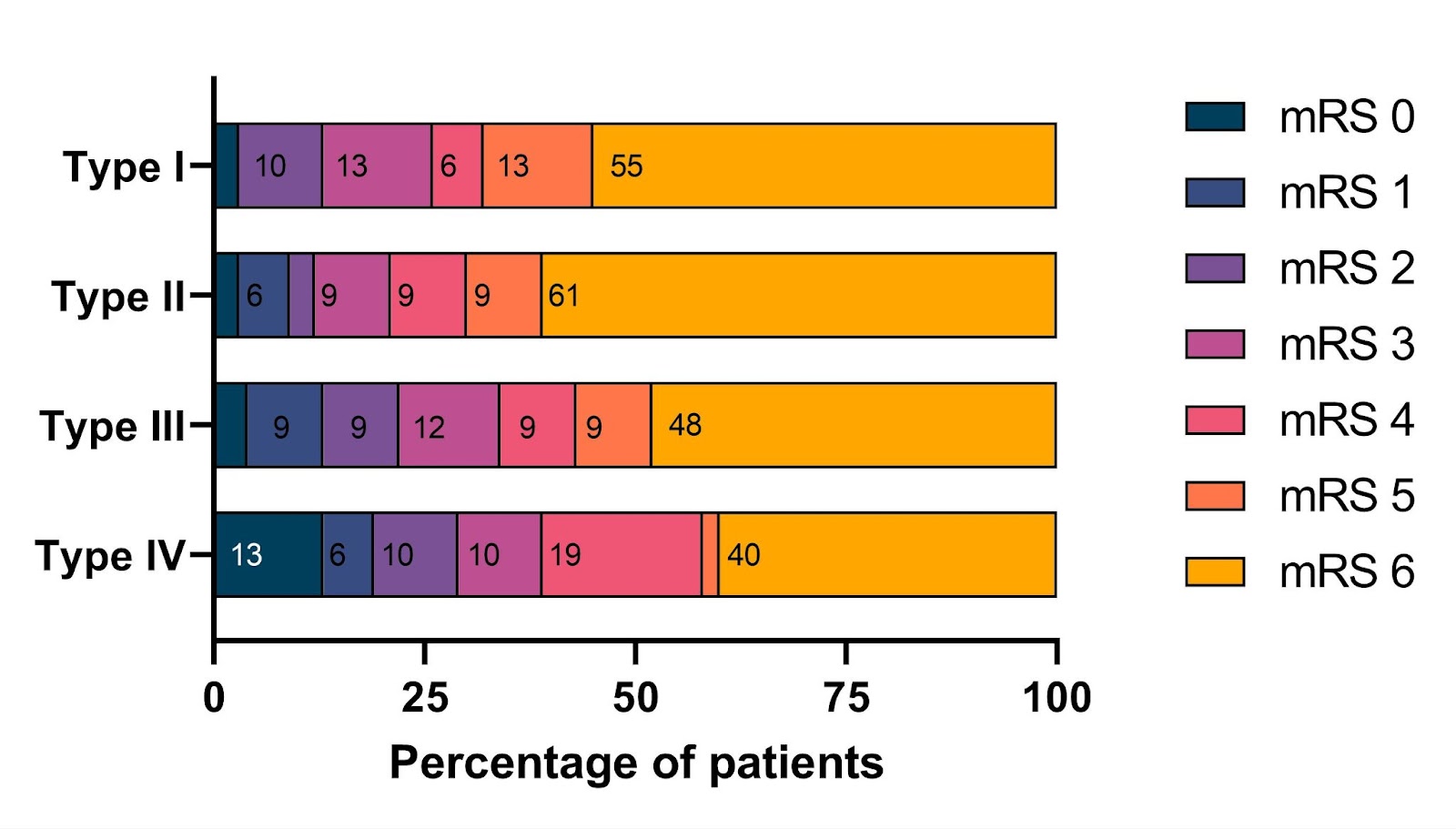


**Figure 3.** Distribution of clinical outcome 90 days post-stroke on the modified Rankin scale (mRS) according to basilar artery occlusion type. Type I are proximal, type II middle, type III distal and type IV tip of the basilar artery occlusions.

|  | **Mean rank diff.** | **significant** | **adjusted p-value** |
| --- | --- | --- | --- |
| Proximal vs middle | 12.54 | no | >0,9999 |
| **Proximal vs distal** | -43.51 | yes | 0.0005 |
| **Proximal vs tip** | -46.65 | yes | 0.0002 |
| **Middle vs distal** | -56.06 | yes | <0,0001 |
| **Middle vs tip** | -59.19 | yes | <0,0001 |
| Distal vs tip | -3.139 | no | >0,9999 |

**Table 1.** Dunn’s multiple comparisons test of differences in stroke etiology

|  | **Odds Ratio** | **Confidence Interval** | **p-value** | **sig** |
| --- | --- | --- | --- | --- |
| Sex | 0,7647 | 0,4004 - 1,458 | 0,4144 | ns |
| Age [years] | 0,9678 | 0,9429 - 0,9921 | **0,011** | * |
| Transfer to center from external clinic | 1,219 | 0,6398 - 2,346 | 0,5481 | ns |
| i.v. thrombolysis | 1,226 | 0,6397 - 2,347 | 0,5382 | ns |
| NIHSS admission | 0,9304 | 0,9021 - 0,9572 | **<0,0001** | **** |
| Pre-stroke mRS | 0,6623 | 0,4845 - 0,8826 | **0,0068** | ** |
| Diabetes | 0,5497 | 0,2297 - 1,213 | 0,1546 | ns |
| Arterial hypertension | 1,08 | 0,4524 - 2,785 | 0,8673 | ns |
| Coronary heart disease | 0,4911 | 0,2199 - 1,031 | 0,0691 | ns |
| Atrial fibrillation | 0,7724 | 0,3929 - 1,494 | 0,447 | ns |
| Dyslipidaemia | 0,7937 | 0,3875 - 1,581 | 0,5173 | ns |
| Wake-up stroke | 0,429 | 0,2026 - 0,8647 | **0,0215** | * |
| pcASPECTS on first imaging (CT or MRI) | 1,159 | 0,9510 - 1,436 | 0,1579 | ns |
| Time from symptom onset to final recanalization status [min] | 1 | 0,9998 - 1,000 | 0,5445 | ns |
| Time from symptom onset to groin puncture [min] | 1 | 0,9998 - 1,000 | 0,4743 | ns |

**Table 2.** Results of the binary univariate logistic regression analysis of favorable outcome including clinical variables.
